# Measuring capabilities in health and physical activity promotion: a systematic review

**DOI:** 10.1186/s12889-020-10151-3

**Published:** 2021-02-15

**Authors:** M. Till, K. Abu-Omar, S. Ferschl, A. K. Reimers, P. Gelius

**Affiliations:** Department of Sport Science and Sport, Friedrich-Alexander-University, Erlangen Nuremberg, Gebbertstraße 123b, 91058 Erlangen, Germany

**Keywords:** Capability approach, Public health, Measurement, Operationalization, Physical activity, health outcomes, Questionnaires

## Abstract

**Background:**

The capability approach by Amartya Sen and Martha Nussbaum has gained increasing attention in the field of public health. As it combines individual, social and structural factors and shifts the focus of attention from the actual behavior towards available options for health behaviors that people can actually choose from, it may help advance our understanding of complex health issues.

**Objectives:**

The aim of this article is to identify and describe tools available to measure capabilities within the context of health, with a specific focus on capabilities for health-enhancing physical activity.

**Method:**

We conducted a systematic literature review using 11 databases covering scientific journal articles published in English or German between the years 2000 and 2020 with a focus on capabilities for health or physical activity.

**Results:**

We found a total of 51 articles meeting our inclusion criteria. Four articles measured capabilities using qualitative methods, one combined qualitative and quantitative methods, while the rest used quantitative methods. We identified a total 11 different capability questionnaires, all showing moderate to good validity/reliability. Only one questionnaire and one interview-based tool specifically dealt with capabilities for health enhancing physical activity.

**Conclusion:**

Although we were able to identify measurement tools for capabilities in health, this review has shown that there is no generic tool available for the measurement across all population- and age-groups, and tools focusing on physical activity are scarce. However, our results can be used as guide for future projects that aim at measuring capabilities.

## Background

Over the last years, the capability approach – originally developed by Amartya Sen [1] in welfare economics – has gained increasing attention in the field of health and has been used in multiple health promotion projects [2–6]. A recent review by Helter et al. [7] highlights this growing relevance of the capability approach in health promotion, particularly regarding its use within health economic evaluation of projects. The capability approach shifts the focus of attention from an individual’s actual behavior – the realization of “various things a person may value being or doing” [8], e. g. having a healthy diet (called “achieved functionings”) – towards the real opportunities – “various combinations of functionings that the person can achieve” [8] (called “capabilities”) – available to individuals to choose from.

The shift of focus from people’s behavior towards their real opportunities, that they can value and realize, can be particularly beneficial in the field of health promotion. In the context of this paper, we look at the capability approach from the perspective of physical activity (PA). PA is commonly defined as “any bodily movements produced by skeletal muscles that result in energy expenditure” [9] and has been generally proven to have a positive impact on people’s health, e. g. in relation to obesity, non-communicable diseases (e. g. diabetes, high-blood pressure), cardio-respiratory health, cancer, mental health and all-cause mortality [10, 11]. Health-enhancing PA (HEPA) may come in many forms and shapes across multiple domains, e. g. during leisure time (e. g. sports, walks or hiking), at the workplace, during transport (e. g. biking to school), or at home (e. g. gardening) [12].

Current efforts to promote PA, however, tend to focus on “downstream” interventions (e. g. physical education in school or structured PA classes for older people) that promise to have immediate effects on the target group’s health [13]. However, such interventions focus mainly on outcome improvement, i. e. achieved health functionings, and tend to neglect the environmental or social components that led to the outcome in the first place. In doing so, such interventions may be less sustainable than more “upstream” interventions whose effects cannot immediately be measured in terms of target group behavior change (e. g. those that initiate infrastructure change [14] or that increase individuals’ physical literacy, i. e. their “motivation, confidence, physical competence, knowledge and understanding to value and take responsibility for maintaining purposeful physical pursuits/activities throughout the lifecourse” [15]). To achieve sustainable behavior change, there is a need to extend the focus of HEPA interventions from focusing solely on outcomes (e. g. steps, hours spent being physically active, effects on weight etc.) towards also considering the capabilities of target groups to engage in desired behavior or to achieve valued states of being.

The capability approach may help achieve this shift of focus by pointing to the benefits in terms of capabilities for healthy behavior [2] or, as in our specific case, HEPA. It explicitly respects people’s freedom to decide for or against a healthy behavior and looks at available or unavailable components which may have led to the specific outcome. Therefore, applying the capability approach within a health promotion project may enhance the target group’s compliance by focusing on how to positively change the opportunities for health that they consider meaningful and desirable, rather than merely “forcing” them to behave in a healthy manner (e. g. through mandatory physical education in schools) to achieve positive change of health outcome.

In general, a person’s capabilities for health enhancing behavior can be assumed to be based on a set of capitals or resources [5] that are “translated” into capabilities through three sets of *conversion factors* [6]: (1) individual (e. g. physical condition, biological health or health literacy), (2) social (e. g. norms and values, social practices or political rules), and (3) environmental factors (e. g. climate, pollution, infrastructure). However, operationalizing a concept as complex as the capability approach [3] (or, to give another example, Antonovsky’s [16] “sense of coherence”) for actual measurement is challenging, as it is rather theoretical in nature and underspecified (potentially by design) with respect to empirical application. Nonetheless, the increasing popularity of the capability approach in health and PA promotion obliges us to assess not only health status and indicators of behavior but also the available opportunities that people have to realize healthy behavior.

The aim of this paper is to support researchers in health and HEPA promotion who intend to use the capability approach by (1) systematically identifying all currently available tools to measure capabilities for health, well-being, and PA, (2) providing an overview of the main features of these tools as well as their psychometric properties, and applicability to different areas, and (3) discussing how the identified capability measures can be specifically used in the field of HEPA promotion by future researchers.

## Methods

Research for this paper was conducted in the context of Capital4Health, a research consortium funded by the German Federal Ministry of Education and Research [01EL1421A-F] which aimed at promoting active lifestyles in four different settings across the life-course using the capability approach. A project (CAPCOM, [01EL1421A]) tasked with fostering cooperation in the consortium conducted the systematic review at hand in order to strengthen its common methodological base. The presented work followed the Preferred Reporting Items for Systematic-Reviews and Meta-Analyses (PRISMA) guidelines [17].

An initial exploratory search for instruments to measure capabilities specifically for PA indicated that only a limited number of instruments were dedicated to this topic, we therefore decided to broaden the search to include capability measurement tools for health in general. This expansion may seem radical but was a logical next step given our health-centered perspective on PA and HEPA [12]. As options for measuring capabilities for PA are limited, gathering information on available measurement tools for the general capabilities of health and well-being will enable the identification of tools that can be adapted to PA or, in cases where adaptation is difficult, provide valuable lessons for the future development of new specific capability measurement tools for HEPA.

Supported by a university librarian, research team developed a set of search strings consisting of variations of the terms “capability approach”, “measurement”, “health” and “physical activity” combined with Boolean operators. A full version of the search term is provided in the appendix. On 14^th^ of October 2020, searches were conducted on the following databases: APA Psycinfo, Psychology and Behavioral Sciences Collection, SPORTDiscus, and APA PsycArticles via EBSCOhost, Applied Social Science Index & Abstracts, Sociological Abstracts, Social Services Abstracts, Worldwide Political Science Abstracts, International Bibliography of the Social Science, and the Sports Medicine & Education Index via ProQuest, and Pubmed.

Table 1 summarizes the inclusion/exclusion criteria applied to the results. Articles were included if they (a) were published between January 2000 and October 2020; (b) were written in English or German; (c) were scientific journal articles; (d) had a clear focus on the operationalization of the capability approach within the context of health or HEPA; and referred to any (e) population, (f) setting, or (g) country.

**Table 1:** Inclusion and exclusion criteria

Criterion	Inclusion	Exclusion
Time	January 2000 – October 2020	Studies before 2000 and after October 2020
Language	English, German	Any other language
Type of publication	Journal Articles	Scientific papers published outside a journal
Focus of study	• Operationalization of the Capability Approach in terms of Amartya Sen/ Martha Nussbaum • Tool to measure Capabilities for health/heath enhancing physical activity	• Outside context of health • Pure article on theory without operationalization of the capability approach
Study population	Any Population	Nil
Setting	Any Setting	Nil
Country	Any country	Nil

Two researchers independently screened all titles/abstracts based on the inclusion/exclusion criteria and discussed their results to resolve disagreement. Two researchers then independently screened the full texts of all remaining papers and discussed their results to reach consensus on the articles to be included for detailed analysis. In addition, the lead author carried out a supplementary hand search. Results of the latter were double-checked by another researcher. The included final search results were imported into Endnote X9 and analyzed regarding (i) the proposed types of measurement instruments for capabilities, (ii) the development process employed to develop these instruments, and (iii) the empirically tested validity, reliability, and responsiveness of the instruments among different target groups.

For better comparison, in the context of this paper, we rated instrument quality as follows: construct validity was categorized as “good” when correlations with any chosen other instrument had shown to be at least moderate and significant, or when its chi-square analysis had shown to be significant at the 5% level [18]. We only rated the outcomes reported in the respective paper but not the measurement tool used for the comparison. Discriminant validity was rated as “good” when the instrument showed a significant (at least *p* < .01) distinction between different areas. Internal consistency with α > .7 was considered “good” [19], as well as test-retest reliability with a moderate (>.41) Cohen’s kappa [20] or an intraclass-correlation coefficient over .75 [21].

## Results

**Fig. 1:**
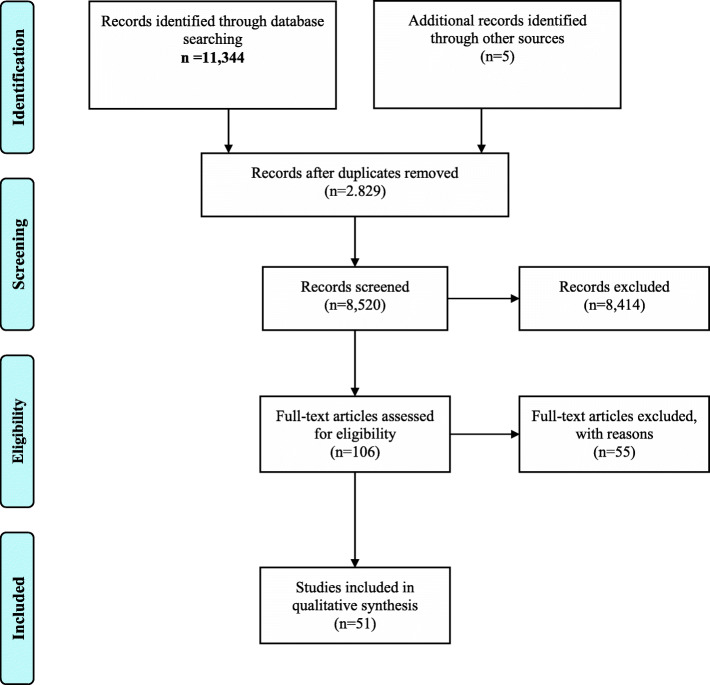
Literature search flow chart based on PRISMA [13]

The search yielded in a total of *N* = 11,354 hits matching the search terms across all eleven databases. After removing all duplicates, a total of 8515 articles remained for screening. Researchers had substantial agreement on title/abstract screening (Cohen’s k = 0.66), disagreeing mostly on the use of the capability approach within a paper [22]. This step yielded a total of 101 articles eligible for full-text screening. Researchers had moderate agreement in full-text screening (Cohen’s k = 0.44) [20], leading to the exclusion of another 55 articles. Disagreement on inclusion or exclusion was mostly about the level of operationalization of the capability approach in papers, i. e. whether articles actually provided a full-fledged measurement tool or merely a theoretical framework. Five additional articles were identified during hand search, resulting in a total of *N* = 51 articles included in this review, covering either the development of instruments for measuring capabilities according to the capability approach or psychometric properties. A visual representation of the search is shown in Fig. 1 using the PRISMA-flowchart [17].

### Types of measurement instruments

Table 2 provides an overview of the different measurement tools reported in the 52 identified articles. We found that instruments to assess capabilities fall into three major categories: (1) qualitative tools, e. g. using interviews or videography (*n* = 5), (2) quantitative tools, e. g. questionnaires (*n* = 46), and (3) mixed method approaches using a combination of interviews and questionnaires (*n* = 1).

**Table 2:** Distribution of measurement tools

			**No.**		
**Capability Measurement Tools**	**Qualitative tools**		1	Interviews [23–26]	
			2	Videography [27]	
	**Mixed-Method**		3	Questionnaire and Interviews [28]	
	**Quantitative tools**		4	Questionnaire combinations (secondary data) [21, 28–30]	
			*5*	*ICECAP*	*ICECAP-O* [31]*
			*6*		*ICECAP-A* [32]*
			*7*		*ICECAP-SCM* [33]*
			*8*		*ICECAP-FC* [34]*
			*9*	*OCAP*	*OCAP* [35]*
			*10*		*OCAP-18* [29]*
			*11*		*OXCAP-MH* [36]*
			*12*	*CQ-CMH*	*CQ-CMH* [37]*
			*13*		*ACQ-CMH* [38]*
			*14*	*Capability-based questionnaire –well-being in patients with chronic pain* [39]*	
			*15*	*CADA* [40]*	

*Note: This table only indicates the articles reporting on the development of the respective tool. *ICECAP-O* ICEpop CAPability measure for older people, *ICECAP-A* ICEpop CAPability measure for adults, *ICECAP-FC* ICEpop CAPability and Functioning measure, *ICECAP-SCM* ICEpop CAPability measure for supportive care, *OCAP* Oxford Capability Questionnaire, *OXCAP-MH* Oxford Capability Questionnaire for Mental Health, *CQ-CMH* Capability Questionnaire for Community Mental Health

In the quantitative category, n = 5 articles measured capabilities through analyzing secondary data (e. g. data from the British Panel Household Survey [41]), while *n* = 41 covered a total of eleven individual questionnaires. Of these, four belong to the *ICECAP*-family (ICEpop CAPability index of the “Investigating Choice Experiments for the Preferences of Older People” project) and use varying sets of items to cover specific target groups and outcome variables: the *ICECAP-O* for older adults [31] and *ICECAP-A* for adults [32] with five items each, the *ICECAP-SCM* measuring capabilities of people in need of supportive care [33] containing seven items, and the *ICECAP-FC* for adults measuring both functioning and capabilities [34] with ten items. Another set of questionnaires comes from the “Oxford Capability Questionnaire” family, including the original *OCAP* (Oxford capability Questionnaire) [35] with 64 items, the shortened *OCAP-18* [42] (18 items), and a version adapted to mental health, the *OxCAP-MH* (Oxford Capability Questionnaire for Mental Health) [36] (16 items). The most comprehensive questionnaires are the *CQ-CMH* (Capability Questionnaire for community mental health) [37] with 104 items and its adapted version, the *ACQ-CMH* (Achieved Capability Questionnaire for community mental health) [38] with 98 items. The systematic search further identified two questionnaires that did not belong to a larger “family” of tools, the *Capability Based Questionnaire for Patients with Chronic Pain* [39] (8 items) and the *Capability Assessment for Diet and Activity* (CADA) geared at adults suffering from obesity and diabetes [40]. All identified questionnaires are constructed for self-completion and use subjective measures to assess capabilities.

### Main aims and methods employed

Table 3 reports on the main aims of the included articles as well as the main methods used to develop the individual measurement tool or to empirically test its measurement properties. Out of the 52 included articles, 8 described the development of a measurement instrument [31–35, 39, 40, 42], 20 focused on checking psychometric properties of existing tools [49, 50, 52, 54–58, 61, 63–71, 74], 2 evaluated different instruments comparatively [28, 75], and 8 reported results of actual measurements of health-related capabilities [23, 24, 27, 41, 44–47]. The remaining (*n* = 14) articles had a mixed focus: on development/measurement (*n* = 2) [25, 26], development/psychometric properties (*n* = 9) [29, 30, 36–38, 43, 51, 53, 62], or comparison/psychometric properties (*n* = 3) [59, 60, 73].

**Table 3:** Description of included studies and tools

*Qualitative tools*								
**No.**	**Tool**	**No. of items**	**Author (year) country**	**Study Aim**	**Focus of tool**	**Language of tool**	**Target population**	**Method**
1.	Interview	8	Weaver et al., (2014) [23], Canada	M	Health and diabetes self-management	English	Adults with diabetes	Measurement via semi-structured interviews; Analysis via two researchers
		n.a.	Ndomoto et al. (2018) [24], UK	M	Health	English	Adults living in rural Kenya and urban deprived UK	Measurement via key informant interviews; *FG* and participant observation.
		10	Sauter et al. (2018) [25], Germany	D/M	Health enhancing PA	German/English	Older adults living in senior residences	Development of interview-guide by *RT* based on Anand’s capability questionnaire [35] and literature on older adult’s physical activity; Measurement via semi-structured interviews
		26/21	Chakraborty et al. (2020) [26]	D/M	Healthy children’s growth	Bangla/English	children living in hoar region of Bangladesh	Development of FG and individual interview guide by *RT* based on literature review and pilot testing; Measurement via FG and in-depth interviews with parents
2.	Videography	n.a.	Petros et al. (2016) [28], USA	M	Mental health recovery	English	Adults with mental illness	Four-week measurement via videography on the topic *Tell us about your recovery*; No *RT* present during recording; Transcription and analysis of data by *RT*
*Mixed method*								
**No.**	**Instrument**	**No. of items**	**Author (year) country**	**Study Aim**	**Focus of Tool**	**Language of tool**	**Target population**	**Method**
3.	Questionnaire and Interview	20	Bucki et al. (2016) [43], Luxembourg	C	Health	Luxembourgish, Portuguese, French, German	Adult care givers	Measurement of relations between health capability factors of care givers using questionnaire-based (HCFC-8) interviews. Statistical analysis using Monte Carlo Markov Chain algorithms.
*Quantitative tools*								
**No.**	**Instrument**	**No. of items**	**Author (year) country**	**Study Aim**	**Focus of Tool**	**Language of tool**	**Target population**	**Method**
4.	Questionnaires used in secondary data	n.a.	Abu-Zaineh & Woode (2018) [44], France	M	Health and self-management	English	Young adults living in Palestine	Measurement of capabilities (health awareness, knowledge and living conditions) via Exploratory Structural Equation Modelling using data from the Palestinian Family Survey.
		n.a.	Anand et al. (2005) [41], UK	M	General WB	English	Adults living in British households	Measurement of capabilities and well-being by regression using data of the British Household Panel Survey
		n.a.	Douptcheva et al. (2014) [45], UK	M	Health	English	Women living in Accra	Measurement of capabilities and functionings to identify factors that influence our health using data from the Women’s Health Study of Accra – Wave II.
		1760	Tellez et al. (2016) [46], France	M	WB	French	Older adults	Measurement of capabilities (freedom to perform self-care activities, freedom to participate in life of the household) by use of a latent variable modelling framework analyzing the 2008 Disability and Health Household Survey of France.
		n.a.	Zwierzchowski and Panek (2020) [47], Poland	M	Subjective WB	Polish	General population ≥ 16	Measurement of capabilities and subjective well-being using the multiple indicator multiple cause model on the European-Survey of Income and Living Conditions in Poland (2015)
5.	ICECAP/ ICECAP-O	5	Coast et al. (2008) [31], UK	D	General WB	English	Adults ≥65	Lay terms defined by *RT* based on in-depth interviews [48]. Iterative semi-structured interviews to ensure understandable language. Valuation via survey interviews.
		5	Coast et al. (2008) [49], UK	PP	General WB	English	Adults ≥65	Validation via Chi-square analysis against socio-demographic information, health, nature of locality and environment, social support, participation, and comparison of data to priori set *RT*-expectations
		5	Flynn et al. (2011) [50], UK	PP	General WB	English	Adults ≥65	Construct validity measurement of tariff scores (Comparison with qualitative interviews of attribute development [51] and subjective wellbeing literature)
		5	Couzner et al. (2012) [52], Australia	PP	General WB	English	Adults ≥65	Measurement of relationship of ICECAP-O to EQ-5D and CTM-3 through Spearman’s rho, *t*-tests and chi-square tests.
		5	Makai et al. (2012) [53], Netherlands	D/PP	General WB	Dutch	Adults ≥65	Forward-backward-translation into Dutch by two independent translators; Measurement of concurrent (correlations of the nursing and family version with EQ-5D, EQ-VAS, Cantril’s ladder, overall life satisfaction) and discriminant validity (chi-square and Mann-Whitney *U* tests*)*
		5	Davis et al. (2013) [54], Canada	C/PP	General WB	English	Adults ≥65	Comparison against the EQ-5D using EFA
		5	Makai et al. (2013) [55], Netherlands	PP	General WB	Dutch	Adults ≥65	Measurement of convergent (correlation with EQ-5D, IADL, GDS-15, SPF-IL and Cantril’s ladder) and discriminant validity (*t* test, one-way ANOVA and stepwise regression analyses)
		5	Horwood et al. (2014) [56], UK	PP	General WB	English	Adults ≥65	Face-validity measurement via “think aloud” study analysis and frequency of participant’s problems
		5	Hörder et al. (2016) [57], Sweden	PP	General WB	Swedish	Adults ≥65	Test-retest reliability (1–2 weeks apart) and item relevance measure (participants rated items from 0 to 100)
		5	Davis et al. (2017) [54], Canada	PP	General WB	English	Adults ≥65	Measurement of responsiveness (regression on age, sex, and faller status)
		5	Sarabia-Cobo et al. (2017) [58], Spain	PP	General WB	Spanish	Adults ≥65	Measurement of construct (factor analysis) and convergent validity (correlation with dimensions of the EQ-5D + C, ADRQL, ADL), and reliability (internal consistency-Cronbach Alpha)
		5	Franklin et al. (2018) [59], UK	C/PP	General WB	English	Adults ≥65	Comparison of (1) tariff scores using OLS and CLAD regression models and (2) domain scores using MNL regression against the EQ-5D-3L
		5	Milte et al. (2018) [60], Australia	C/PP	General WB	English	Adults ≥65	Comparison against the EQ-5D-3L using Spearman correlation coefficient and multiple linear regression
		5	Mitchell et al. (2020) [61], UK	PP	General WB	English	Adult ≥65	Measurement of response validity among people requiring kidney care using a think-aloud study
		5	Baji et al. (2020) [62], Hungary	D/PP	General WB	Hungarian	Adult ≥65	*RT* translated original version into Hungarian; forward-backward translation; interviews (*n* = 15) to assess comprehensiveness and relevance; Measurement of: construct validity (one-way subgroup comparison and regression analysis); convergent validity (Pearson’s and Spearman’s correlation (with EQ-5D-5L, EQ VAS, WHO-5; happiness and satisfaction VAS,SWLS); Test-retest reliability (ICC baseline and 5% of participants right after baseline
6.	ICECAP-A	5	Al-Janabi et al. (2012) [32], UK	D	General WB	English	Adults ≥18	Identification of important components of life through in-depth interviews; Iterative semi-structured interviews to refine attributes to a self-completion measure with one item per attribute
		5	Al-Janabi et al. (2013) [63], UK	PP	General WB	English	Adults ≥18	Think-aloud and semi-structured interviews to assess the feasibility of a self-reporting capability measurement
		5	Al-Janabi et al. (2013) [64], UK	PP	General WB	English	Adults ≥18	Measurement of construct validity (univariate analysis and correlations based on hypotheses made in advance)
		5	Al-Janabi et al. (2015) [65], UK	PP	General WB	English	Adults ≥18	Measurement of test-retest reliability (ICC- baseline and 2-week capability index scores)
		5	Keeley et al. (2015) [66], UK	PP	General WB	English	Adults ≥18	Measurement of responsiveness (anchor-based analysis; anchors: EQ-5D-3L, GAD-7, PHQ-8)
		5	Goranitis et al. (2016) [67], UK	PP	General WB	English	Adults ≥18	Measure of acceptability, construct validity (convergent: Pearson’s correlation with EQ-5D-3L and ICIQ-OAB, Spearman’s correlation coefficient across dimension scores, and index and dimension scores; discriminant: one-way ANOVA and Kruskal-Wallis *H* test)
		5	Goranitis et al. (2016) [68], UK	PP	General WB	English	Adults ≥18	Assessment of construct validity (convergent: Pearson’s correlation with EQ-5D-5L; Discriminant: univariate and multivariate analysis) and sensitivity to change
		5	Mitchell et al. (2017) [69], UK	PP	General WB	English	Adults ≥18	Concept-mapping from condition-specific and capability items; Discriminant validity testing (Mann-Whitney *U* test using DASS-D and K10 data; Multivariable regression analysis using OLS)
		5	Linton et al. (2018) [70], Germany	PP	General WB	German	Adults ≥18	Measurement of internal-consistency (Cronbach’s Alpha), convergent (Pearson’s correlation with EQ-5D-3L, SF-6D, SWLS scores), and construct validity (OLS regressions)
		5	Tang et al. (2018) [51], China	D/PP	General WB	Chinese	Adults ≥18	*RT* translated original version into Chinese; *FG* evaluated appropriateness of the translation; pilot testing; backward translation; online-survey to check acceptability, reliability (item correlations), and validity (EFA and correlations with EQ-5D-3L and EQ-VAS)
		5	Holst-Kristensen et al. (2020) [43], Denmark	D/PP	General WB	Danish	Adult ≥18	*RT* translated original version into Danish; forward-backward translation; pilot-testing in general population
		5	Shahataheri et al. (2020) [29], Iran	D/PP	General WB	Persian	Adult ≥18	*RT* translated original version into Persian; forward-backward translation; pilot-testing in general population; Measurement of test-retest reliability (ICC-baseline and 2-week)
		5	Mah et al. (2020) [71], Canada	PP	General WB	English	Adult ≥18	Measurement of construct validity: discriminant (*t* test, linear trend analysis or multiple regression); convergent (correlation with measures of the same concept: AQoL-8D, EQ-5D-5L, HUI-3, SF- 6D)
		5	Mitchell et al. (2020) [61], UK	PP	General WB	English	Adult ≥18	Measurement of response validity among people requiring kidney care using a think-aloud study
		5	Baji et al. (2020) [62], Hungary	D/PP	General WB	Hungarian	Adult ≥18	*RT* translated original version into Hungarian; forward-backward translation; interviews (n = 15) to assess comprehensiveness and relevance; Measurement of: construct validity (one-way subgroup comparison and regression analysis); convergent validity (Pearson’s and Spearman’s correlation (with EQ-5D-5L, EQ VAS, WHO-5; happiness and satisfaction VAS,SWLS); Test-retest reliability (ICC baseline and 5% of participants right after baseline
7.	ICECAP-SCM	7	Sutton & Coast (2014) [33], UK	D	WB in end of life care	English	People at end of life	Interviews to determine conceptual elements of a good death; follow-up interviews to check conceptual attributes
8.	ICECAP-FC	10	Al-Janabi (2018) [34], UK	D	WB capabilities and functionings	English	Adults ≥18	ICECAP-A modified with additional question on functioning to each attribute by *RT*
9.	OCAP	64	Anand et al. (2009) [35], UK	D	General Capabilities (e.g. enjoying recreational time, political views, making friends bodily health and integrity)	English	Adults ≥18	Development of items based on Nussbaum criteria [72]
10.	OCAP-18	18	Lorgelly et al. (2015) [42], UK	D	General Capabilities (e.g. enjoying recreational time, political views, making friends bodily health and integrity)	English	Adults ≥18	Items, based on OCAP-questionnaire [35], reduced on analysis of *FG*, cognitive interviews, and factor analysis
11.	OxCAP-MH	16	Simon et al. (2013) [36], UK	D/PP	General capabilities for mental health	English	Adults ≥18 with a mental illness	Adaption of the OCAP-18 [20] based on expert-*FG* and validation (correlation with GAF, EQ-5D-VAS, EQ-5D-3L)
		16	Vergunst et al. (2017) [49], UK	PP	General capabilities for mental health	English	Adults ≥18 with a mental illness	Measurement of internal-consistency (Cronbach’s alpha), test-retest (1-week apart; ICC), and construct validity (correlation with EQ-5D, BPRS, GAS, SIX)
		16	Simon et al. (2018) [30], UK	D/PP	General capabilities for mental health	English	Adults ≥18 with a mental illness	Forward-backward-translation of OxCAP-MH into German and linguistic validation through German native speakers
		16	Laszewska et al. (2019) [73], Austria	C/PP	General capabilities for mental health	German	Adults ≥18 with a mental illness	Comparison against the EQ-5D-5L (EFA). Measurement of responsiveness (anchor questionnaires and standardized response mean), discriminant validity (subgroup comparison using *t* test and one-way ANOVA), and test-retest (ICC; baseline - max 30 days after)
12.	CQ-CMH	104	Sacchetto et al. (2016) [37], Portugal	D/PP	Mental Health	Portuguese	Consumers of mental health services	*FG* interview data analysis; development of item/rating scale by steering committee and additional comparison with Nussbaum criteria [34]; Assessment of face-validity
13.	ACQ-CMH-98	98	Sacchetto et al. (2018) [38], Portugal	D/PP	Mental Health	Portuguese	Consumers of mental health services	Adaption of the CQ-CMH questionnaire [37] based on panel members judgement; Measurement of validity (correlation with WHOQOL-Bref, RAS, K6)
14.	Capability-based questionnaire	8	Kinghorn et al. (2015) [39], UK	D	WB	English	People suffering from chronic pain	*FG* interview and individual interviews to identify list of important capabilities; Development of questionnaire for self-completion based on identified capabilities by *RT*
15.	CADA	34	Ferrer et al. (2014) [40], USA	D	Physical Activity and Diet	English	Adults with obesity and diabetes	*FG* interviews were used to identify important themes; questionnaire created by *RT* based on themes

*ADL* activities of daily living, *ADRQL* Alzheimer’s disease related Quality of life, *BPRS* Brief Psychiatric Rating Scale, *C* Comparison, *CTM-3* 3-Item Care Transition Measure, *D* Development, *EFA* exploratory factor analysis, *FG* Focus group; *GAF* Global Assessment of Functioning, *ICC* Intra-class correlation coefficient, *M* Measurement*, OLS* ordinary least square, *RAS* Recovery Assessment Scale, *RT* Researcher Team, *SIX* Objective Social Outcomes Index, *V* Validation, *WB* Well-Being

Among the qualitative tools, only Sauter et al. [25] provided details on the development process: Their interview guidelines were the result of literature screening and a conscious selection of specific items from the *OCAP* questionnaire [35]. The identified questionnaires were developed using different methodologies. For example, the *OPCAP* [35] is based on a set of largely theoretical criteria by Martha Nussbaum, who co-developed the original capability approach [76]. The *ICECAP-O* questionnaire was compiled based on a previously conducted literature review and developed through in-depth interviews with the respective target group [31]. The *ICECAP-A* [32] and *ICECAP-SCM *[33], the *Capability Based Questionnaire for Patients with Chronic Pain* [39], and *CADA* [40] were developed by conducting iterative interviews with the respective target group. The *CQ-CMH* [37] emanated from the analysis of focus group data, expert opinion, and an additional alignment with the Nussbaum criteria.

Articles reporting on the validation of questionnaires used different methodological approaches. Convergent and construct validity were mostly investigated by correlating results with those measured via other questionnaires that measure well-being or health aspects (e. g. EQ-5D) [29, 35, 36, 38, 51, 53, 55, 58, 60, 62, 66–68, 70, 73, 74] or using Chi-Square analysis [31, 52, 64]. Discriminant validity was ascertained by performing uni- or multivariate analysis [29, 55, 58, 67–69, 71, 73]. Some questionnaires were further been checked regarding their reliability using test-retest analysis [29, 38, 43, 57, 62, 73, 74, 77], or regarding their responsiveness via anchor-based analysis [54, 66, 67]. Moreover, the *ICECAP-O* and the *Ox-CAP *questionnaire were evaluated comparatively to the EQ-ED questionnaire by correlating their results [59, 60, 73, 75].

Articles reporting on studies that directly measured capabilities without developing or validating any tools for future use were only found among the qualitative studies and secondary data analyses. Qualitative measurement was performed either by semi-structured interviews [23–26], observation [24] or video analysis [27], while secondary data was analyzed via methods such as regression [41] or equation modelling [44].

### Measurement properties

The major measurement properties of the different tools are shown in Table 4. Sample sizes among the qualitative and mixed methods approaches varied between *n* = 12 [27] and *n* = 64 [26], while numbers were naturally much larger for the secondary data analyses (between *n* = 2814 [45] and *n* = 25,180 [44]). Target groups varied widely, from adults in general [24, 32, 35, 41, 78], children under the age of two [26], young adults [44] and older adults [25, 31, 46], to adults with special conditions or characteristics [23, 27, 36–40] or only women [45].

**Table 4:** Psychometric properties of the identified tools

*Qualitative/mixed methods*							
**No.**	**Instrument**	**Author (year)**	**Sample size, description of study population; age; % male; country**				
1.	Interviews	Weaver et al., (2014) [23]	*n* = 45; adults with diabetes; M = 60; 42%; Canada				
		Ndomoto et al. (2018) [24]	*n* = 55; whole community; n.a.; n.a.; rural Kenya and urban deprived UK				
		Sauter et al. (2018) [25]	*n* = 26; older adults; 65+;38%; Germany				
		Chakraborty et al. (2020) [26]	*n* = 64 in 8 focus-group and 8 in depth interviews; parents of children under 2 years of age; 16+; 42%; Bangladesh				
2.	Videography	Petros et al. (2016) [28]	*n* = 12; adults with mental illness; n.a.; n.a.; USA				
*Mixed method*							
**No.**	**Instrument**	**Author (year)**	**Sample size, description of study population; age; % male; country**				
3.	Questionnaire and Interviews	Bucki et al. (2016) [43]	*n* = 62; adult care givers; M = 59; 36%; Luxembourg				
*Quantitative*							
**No.**	**Instrument**	**Author (year)**	**Sample size, description of study population; age; % male; country**	**Validity**		**Reliability/ Responsiveness/ Sensitivity**	
4.	Secondary Data Analysis	Abu-Zaineh & Woode (2018) [44]	*n* = 25,180; young adults: M = 21; 50%; Palestine	n.a		n.a.	
		Anand et al. (2005) [41]	*n* = 12,040; adults; 18+; 45%; UK	n.a.		n.a.	
		Douptcheva et al. (2014) [45]	*n* = 2814; women; 18+; 0%; Ghana	n.a.		n.a.	
		Tellez et al. (2016) [46]	*n* = 8841; older adults; 60+; n.a.; France	n.a.		n.a.	
		Zwierzchowski and Panek (2020) [47]	*n* = 25,830; adults; 16+; n.a.; Poland	n.a.		n.a.	
5.	ICECAP/ ICECAP-O	Coast et al. (2008) [31]	*n* = 255; older adults; 65+; 56%; UK	n.a.		n.a.	
		Coast et al. (2008) [49]	*n* = 314; older adults; 65+; 54%; UK	***Construct:*** EQ-5D overall value and Attachment χ^2^ = .42 Security χ^2^ = .008 (*p* < .01) Role χ^2^ = <.001 (*p* < .01) Enjoyment χ^2^ = <.001 (*p* < .01) Control χ^2^ = <.001 (*p* < .01)		n.a.	
		Flynn et al. (2011) [50]	*n* = 809; older adults; 65+; 49%; UK	***Construct:*** comparison ICECAP-O tariff scores with qualitative interviews of attribute development [51] and subjective wellbeing literature provides construct validity.			
		Couzner et al. (2012) [52]	*n* = 82; older adults in rehabilitation; M = 76; 50%; Australia	***Construct:*** EQ-5D overall value and Attachment χ^2^ = .741 Security χ^2^ = .088 Role χ^2^ = .092 Enjoyment χ^2^ = .058 Control χ^2^ = .043 (*p* < .05) ICECAP-O and CTM-3 Spearman’s *r* = .23; (*p* < .05)and EQ-5D Spearman’s *r* = .44; (*p* < .001)		n.a.	
		Makai et al. (2012) [53]	*n* = 122; older adults; M = 82; 32%; Netherlands	***Convergent:*** ICECAP-O and nursing version of EQ-5D *r* = .48 (*p* < .001) Overall life *r* = .52 (*p* < .001)	ICECAP-O and family version of EQ-5D *r* = .57 (*p* < .001) Overall life *r* = .48 (*p* < .001)	n.a.	
		Davis et al. (2013) [54]	*n* = 215; older adults post falls; M = 79; n.a.; Canada	***Construct:***Two factor analysis indicated two separate but correlated factors, supporting that the instruments provide complementary data with RMSEA (90% CI) = .05 (.00–.09)		n.a.	
		Makai et al. (2013) [55]	*n* = 275; older adults post hospitalization; 65+; 46%; Netherlands	***Convergent:*** Correlation ICECAP-O significant to Cantril’s ladder *r* = .51(*p* < .001) SPF_IL *r* = .60(*p* < .001) EQ-5D *r* = .40(*p* < .001) SF-20 *r* = .47(*p* < .001)	***Discriminant:*** EQ 5D Top 50% M = .90 (*p* < .01) Bottom 50% M = .80 Multimorbid Max. 1 chronic condition M = .89 (*p* < .01) More than 2 conditions M = .82	n.a.	
		Horwood et al. (2014) [56]	*n* = 20; older adults with hip/knee replacement; M = 70; 30%; UK	***Face:*** Majority of participants had no problems completing the measure		n.a.	
		Hörder et al. (2016) [57]	*n* = 40; older adults; 70; 48%; Sweden	n.a.		***Test-retest*:** ICC = .80 systematic disagreement Cohen’s κ (95% CI) Attachment κ = .34; −.17 (−.35 – -.03) (significant) Security κ = .22; .05 (−.11–.20) Role κ = .41; .00 (−.16–.16) Enjoyment κ = .24; −.02 (−.19–.14) Control κ = .17; −.13 (−.32–.05)	
		Davis et al. (2017) [54]	*n* = 247; older adults with impaired mobility; 80 ± 7; 37%; Canada	n.a.		***Responsiveness:*** Change Baseline to 12-Month follow up: M = −.016 (*p* < .05); *r* = .50 Relation of change divided by faller status: Change Baseline to 12-Month follow up, faller: M = -.13 Change Baseline to 12-Month follow up, non-faller: M = .00	
		Sarabia-Cobo et al. (2017) [58]	*n* = 217; older adults with dementia; M = 87; 19%; Spain	***Convergent:*** Correlation EQ-5D + C to ICECAP-O tariff: *r* = .62 (*p* < .01) Attachment *r* = .11 (*p* < .05) Security *r* = .32 (*p* < .05) Role *r* = .71 (*p* < .01) Enjoyment *r* = .56 (*p* < .01) Control *r* = .41 (*p* < .01)	***Discriminant:*** Depression severity Mildmean M = .72 (*p* < .01) Moderate M = .63 Severe M = .50 Care level Low M = .70 (*p* < .01) Medium M = .59 High M = .39	***Internal consistency:*** α = .820	
		Franklin et al. (2018) [59]	*n* = 584; older adults; 65+; 38%; UK	***Construct:*** OLS model with EQ-5D-3L items as discrete variables, including age, sex and care home explanatory variables produced best overall model: RMSE = .16; *R*^*2*^ = .35		n.a.	
		Milte et al. (2018) [60]	*n* = 87; older adults following a hip fractur; 60+; 30%; Australia	***Convergent:*** Spearman Correlation EQ-5D-3L scores to (95% CI; *p*-values) Attachment: *r* = .27 (.07–.43; .013) Security: *r* = .51 (.32–.67; <.001) Role: *r* = .34 (.12–.52; .002) Enjoyment: *r* = .26 (.03–.46; .016) Control: *r* = .46 (.23–.62; .000)		n.a.	
		Mitchell et al. (2020) [61]	*n* = 30; adults requiring kidney care; 18+; 77%; UK	***Process:*** Total Errors/Struggles in Areas during Think-aloud study Attachment: 2 Security: 1 Role: 5 Enjoyment: 2 Control: 0		n.a.	
		Baji et al. (2020) [62]	*n* = 453; older adults; 65+; 50.1%; Hungary	***Convergent:*** Pearson’s Correlation ICECAP-O scores to EQ-5D-5L *r* = .65 EQ-VAS *r* = .50 Happiness-VAS *r* = .52 Satisfaction with life *r* = .57 WHO-5 *r* = .61 SWLS *r* = .52		***Internal-consistency*:** Cronbach’s α = .86 ***Test-retest:*** ICC = .97 (95% CI, .94–.98) Attachment 96.2% Security 96.2% Role 90.6% Enjoyment 94.3% Control 96.2%	
6.	ICECAP-A	Al-Janabi et al. (2012) [32]	*n* = 36; adults; 18+; 41%; UK	n.a.		n.a.	
		Al-Janabi et al. (2013) [63]	*n* = 34; adults; 18+; 47%; UK	***Face:*** Individuals largely responded to questions in intended manner and encountered problems on fewer than 10% of the items.		n.a.	
		Al-Janabi et al. (2013) [64]	*n* = 418; adults; M = 51.7; 38%; UK	***Construct:*** Associations between EQ-5D and Stability χ^2^ = <.001 (*p* < .01) Attachment χ^2^ = .34 Autonomy χ^2^ = <.001 (*p* < .01) Achievement χ^2^ = <.001 (*p* < .01) Enjoyment χ^2^ = <.001 (*p* < .01)		n.a.	
		Al-Janabi et al. (2015) [65]	*n* = 237; adults; 18+; 52%; UK	n.a.		***Test-retest*:** Stability 89.8%; κ = .61 Attachment 88.8%; κ = .57 Autonomy 87.8%; κ = .52 Achievement 88.1%; κ = .53 Enjoyment 88.1%; κ = .54 ICC = .72	
		Keeley et al. (2015) [66]	*n* = 357; adults with knee pain; M = 64; 49%; UK	***Construct:*** Correlation ICECAP-A to EQ-5D-3L *r* = .255 GAD-7 *r* = −.205 PHQ-8 *r* = −.190		***Responsiveness:*** Anchor-based analysis (baseline and 6-months follow-up) Mean ICECAP-A change (95% CI) EQ-5D-3L Improved .02 (.002–.042) (*p* < .05) EQ-5D-3L no change −.003 (−.128–.007) EQ-5D-3L worsened −.54 (−.084–-.024) (*p* < .01) GAD-7 Improved .020 (.002–.042) GAD-7 no change -.004 (−.003–-.011) GAD-7 worsened -.07 (−.11–-.032) (i < .01) PHQ-8 Improved .014 (−.005–.032) PHQ-8 no change .003 (−.006–.011) PHQ-8 worsened -.048 (−.078–-.017) (*p* < .01)	
		Goranitis et al. (2016) [67]	*n* = 478; women with irritative lower urinary tract syndrome; M = 55; 0%; UK	***Convergent:*** EQ-5D correlated (*p* < .01) to Stability *r* = .38 Attachment *r* = .21 Autonomy *r* = .48 Achievement *r* = .45 Enjoyment *r* = .40 ICIQ-OAB correlated (*p* < .01) to Stability *r* = −.23 Attachment *r* = −.12 Autonomy *r* = −.19 Achievement *r* = −.21 Enjoyment *r* = −.25	***Discriminant*** ICECAP-A mean score (SD) Total impact of symptoms Low M = .86(.14)(*p* < .01) Moderate M = .87 (.13) High M = .81 (.18)	***Responsiveness:*** ICECAP-A Score change baseline to follow-up (SD) Symptoms’ bother Increased bother -.05 (.15) (*p* < .01) Same bother -.03 (.17) Lower bother .00 (.15) Symptoms’ frequency Improved .00 (.15) Same level -.039 (.13) Deteriorated .06(.18) (*p* < .01)	
		Goranitis et al. (2016) [68]	*n* = 83; adults with opiate dependence; M = 37; 87%; UK	***Convergent:*** Correlation of ICECAP-A to Psychological health *r* = .55 (*p* < .01) Physical health *r* = .36 (*p* < .01) Quality of Life *r* = .55 (*p* < .01)	***Discriminant:*** ICECAP-A mean score; ±SD: Psychological health High M = .57; ±.19 (*p* < .01) Low M = .74; ±.15 Physical health status High M = .59; ±.20 (*p* < .01) Low M = .71; ±.17 Overall Quality of life High M = .58; ±.19 (*p* < .01) Low M = .75; ±.14	***Sensitivity:*** ICECAP-A mean change baseline to 3-months follow-up; ±SD: Psychological health Not improved M = .00; ±.19 Improved M = .08; ±.13 (*p* < .01) Physical health status Not improved M = .04; ±.19 Improved M = .05; ±.13 (*p* < .05) Overall Quality of life Not improved M = .02; ±.17 Improved M = .07; ±.15(*p* < .05)	
		Mitchell et al. (2017) [69]	*n* = 617; adults with depression; 18+; 33%; UK		***Discriminant:*** ICECAP-A mean score to DASS-D Normal/well M = .84 Mild M = .71 Moderate M = 0.71 Severe M = .64 Very severe M = .47	n.a.	
		Linton et al. (2018) [70]	*n* = 2501; adults (healthy or with Arthritis, Asthma, Cancer, Depression, Diabetes, hearing problems, heart disease); 18+; 52%; Germany, UK	***Convergent:*** Correlation of ICECAP-A and EQ-5D-5L Germany *r* = .62 UK *r* = .61 SWLS Germany *r* = .66 UK *r* = .68 SF-6D Germany *r* = .64 UK *r* = .65		***Internal-consistency:*** (Cronbach’s α) across subsamples Germany; UK	
						Overall sample α = .83; .85 Healthy α = .78; .80 Arthritis α = .74; .78 Asthma α = .77; .83 Cancer α = .86; .83	Depression α = .78;.79 Diabetes α = .83;.86 Hearing loss α = .74;.84 Heart disease α = .83;.85
		Tang et al. (2018) [49]	*n* = 975; adults; 18+/M = 34; 47%; China	***Construct:***Two factor-analysis indicate a different construct between ICECAP-A and EQ-5D-3L Correlation of ICECAP-A and EQ-5D-3L Stability *r* = .39 (*p* < .01) Attachment *r* = .34 (*p* < .01) Autonomy *r* = .38 (*p* < .01) Achievement *r* = .27 (*p* < .01) Enjoyment *r* = .38 (*p* < .01)		***Internal-consistency*:** Cronbach’s α = .799	
		Holst-Kristensen et al. (2020) [51]	*n* = 332; adults; 18+/M = 57; 55%; Denmark	n.a.		***Test-retest:*** Stability 91.4%; κ = .58 Attachment 90.5%; κ = .66 Autonomy 89.3%; κ = .46 Achievement 91.0%; κ = .57 Enjoyment 90.0%; κ = .60 Individual: ICC = .86 (95% CI .83–.88) Group: ICC = .92 (95% CI .91–.94)	
		Shahataheri et al. (2020) [43]	*n* = 1200; adults; M = 45.6; 45.6%; Iran	***Convergent:*** Polychoric correlation of EQ-5D-5L Scores to: ICECAP-A Scores: *r* = .48 Stability: *r* = .34 Attachment: *r* = .19 Autonomy: *r* = .41 Achievement: *r* = .53 Enjoyment: *r* = .40 EQ-VAS Scores to: ICECAP-A Scores: *r* = .49 Stability: *r* = .39 Attachment: *r* = .28 Autonomy: *r* = .33 Achievement: *r* = .45 Enjoyment: *r* = .40	***Discriminant:*** ICECAP-A mean score; ±SD: Education Primary/ High School M = .64; ±.26 (*p* < .001) Diploma M = .79; ±.15 University Degree M = .81; ±.15 Gender Male M = .76; ±.16 (*p* = .185) Female M = .79; ±.16 EQ-VAS (health Status) < 70 M = .66; ±.18 (*p* < .001) > 70 M = .81; ±.14	***Internal-consistency*:** Cronbach’s α for Capability Index Score α = .82 Stability α = .77 Attachment α = .80 Autonomy α = .81 Achievement α = .77 Enjoyment α = .78 ***Test-retest:*** ICC for Capability Index Score =.90 (95% CI, .89–.91) Stability =.96 (95% CI, .95–.96) Attachment = .94 (95% CI, .93–.95) Autonomy = .93 (95% CI, .92–.94) Achievement = .96 (95% CI, .95–.96) Enjoyment = .95 (95% CI, .95–.96)	
		Mah et al. (2020) [29]	*n* = 364; adults with Spinal Cord Injury; 18+/M = 50.4; 63%; Canada	***Convergent:*** Pearson’s correlation ICECAP-A Scores to: AQoL-8D *r* = .74 EQ-5D-5L *r* = .57 HUI-3 *r* = .50 SF- 6D *r* = .58	***Discriminant:*** Confirmed for constructs (*p* < .001): General Health; Mental Health; Social Functioning; Role/activity limitations; Independence (self-care); Independence (mobility); Life Satisfaction; Secondary Health Conditions; Paid Employment; Happiness; Household Income	n.a.	
		Mitchell et al. (2020) [71]	*n* = 30; adults requiring kidney care; 18+; 77%; UK	***Process:*** Total Errors/Struggles in Areas during Think-aloud study Stability: 2 Attachment: 0 Autonomy: 2 Achievement: 2 Enjoyment: 2		n.a.	
		Baji et al. (2020) [62]	*n* = 1568; adults 18+; 50%; Hungary	***Convergent:*** Pearson’s Correlation ICECAP-A scores to EQ-5D-5L *r* = .57 EQ-VAS *r* = .52 Happiness-VAS *r* = .50 Satisfaction with life *r* = .52 WHO-5 *r* = .53 SWLS *r* = .45		***Internal-consistency*:** Cronbach’s α = .863 ***Test-retest:*** ICC = .94 (95% CI, .90–.97) Stability 85.5% Attachment 95.5% Autonomy 91.5% Achievement 91.5% Enjoyment 93.5%	
7.	ICECAP-SCM	Sutton & Coast (2014) [33]	*n* = 23; older adults; 65+; n.a.; UK	n.a.		n.a.	
8.	ICECAP-FC	Al-Janabi (2018) [34]	*n* = 943; adults with long-term after-effects of meningitis; M = 53; 25%; UK	n.a.		n.a.	
9.	OCAP	Anand et al. (2009) [35]	*n* = 1048; adults; 18+; n.a.; UK	n.a.		n.a.	
10.	OCAP-18	Lorgelly et al. (2015) [42]	*n* = 198 (qualitative), *n* = 1048 (quantitative); adults; M = 46; 63%; UK	***Construct:*** Pairwise correlation with EQ-5D-3L = .576 (*p* < .001)		n.a.	
11.	OxCAP-MH	Simon et al. (2013) [36]	*n* = 333; adults with a mental illness; M = 40; 67%; UK	***Convergent:*** Significant correlation of OxCAP-MH scores with GAF *r* = .25 EQ-5D VAS *r* = .51 EQ-5D-3L *r* = .41		n.a.	
		Vergunst et al. (2017) [49]	*n* = 172; adults with psychosis; M = 38; 72%; UK	***Convergent:*** Correlation of OxCAP-MH with EQ-5D-3L *r* = .452 (*p* < .001) EQ-5D VAS *r* = .522 (*p* < .001) BPRS *r* = −.413 (*p* < .001) GAF *r* = .240 (*p* < .001) SIX *r* = .118		***Internal consistency*** Cronbach’s α = .79 ***Test-retest (1-week apart):*** ICC = .86 (*p* < .001) Adjusted *R*^*2*^ = .73 ***Sensitivity:*** Baseline (T1) M = 67.7 (13.8) 12 months follow up (T2) M = 70.8 (11.85) One-SEM values T1 = 6.47; T2 = 6.49	
		Simon et al. (2018) [30]	*n* = 10; adults with mental illness; *M* = 37; 40%; UK	n.a.		n.a.	
		Laszewska et al. (2019) [73]	*N* = 159; adults with mental illness; *M* = 45; 36%; Austria	***Convergent:*** Correlation of OxCAP-MH change scores with EQ-5D-3L *r* = .30 (*p* < .05) EQ-5D VAS *r* = .31 (*p* < .05) BSI-18 *r* = −.42 (*p* < .05) GAF *r* = .15 (*p* < .05) Mini-ICF-APP *r* = −.10	***Discriminant:*** OxCAP-MH mean score (SD): Multi-morbidity one Axis diagnosis M = 68.2(14.4) ≥2 Axis diagnoses M = 56.0(16.8) (*p* < .001) Rating of QoL Very poor/poor M = 48.0(15.4) Neither poor or good M = 65.3(11.5) (*p* < .001) Good/very good M = 74.3(11.2) (*p* < .001)	***Test-retest*** (after 30 days) Cronbach’s α = .85 ICC = .80 (95%CI .69–.87)	
12.	CQ-CMH	Sacchetto et al. (2016) [37]	*N* = 50; adults with mental illness; M = 42; 70%; Portugal	***Face:*** 15 participants confirmed familiarity with language used and relevance of addressed issues. Questionnaire rated as understandable and easy to fill out but too extensive.		n.a.	
13.	ACQ-CMH-98	Sacchetto et al. (2018) [38]	*n* = 332; adults with mental illness; M = 44; 59%; Portugal	***Content:*** Participants (*n* = 15) CVI: .89 *Convergent:* Pearson’s correlation with WHOQOL-Bref *r* = .60 (*p* < .001) K6 *r* = .46 (*p* < .001)	***Discriminant:*** Pearson’s correlation with RAS *r* = −.17 (*p* = .046)	***Test-retest:*** 55% of items high (*r* = .9 to ≥6) 45% of items low (*r* = < 6) ANOVA test significant for 5 items (*p* < .05) ***Internal consistency:*** Optimism α = .91 Affiliation α = .84 Activism α = .84 Practical Reason α = .76 Self-sufficiency and Self-determination α = .76 Family α = .78	
14.	Capability-based questionnaire	Kinghorn et al. (2015) [39]	*n* = 16; adults with chronic pain; 33+; 43%; UK	n.a.		n.a.	
15.	CADA	Ferrer et al. (2014) [40]	*n* = 109; adults with obesity and diabetes mellitus; M = 49; 22%; USA	n.a.		***Internal-consistency***: Convenience, cost: α = .78 Neighborhood opportunity: α = .78 Barriers: α = .75 Knowledge: α = .83 Time Pressure: α = .75 Family support: α = .62 Spouse/partner: α = .65 Nonfamily support: α = .80	

*BPRS* Brief Psychiatric Rating Scale, *CTM-3* 3-Item Care Transition Measure, *DASS-D* Depression Anxiety Stress Scales, *GAF* Global Assessment of Functioning, *ICC* Intra-class correlation coefficient, *K6* Kessler Psychological Distress Scale, *M* Mean*, OLS* ordinary least square, *PHQ-8* Patient Health Questionnaire depression scale, *RAS* Recovery Assessment Scale, *SEM* Structural equation modeling, *SIX* Objective Social Outcomes Index

Sample sizes for the identified questionnaires varied substantially, ranging from *n* = 10 [30] to *n* = 2501 [70]. For the ICECAP-O, six articles reported on the intended target group of adults over 65 [49, 50, 53, 57, 59, 62]. Other publications also applied it specifically to people with a medical condition [54, 58, 61] or within a rehabilitation context [52, 55, 56, 60]. The *ICECAP-A* was developed to measure capabilities among adults over 18. Six of the identified articles used this target group [29, 32, 43, 51, 63, 64, 77] while others validated it pointedly for adults with knee pain [66], opiate dependence [68], depression [69], among women suffering irritative lower urinary tract syndrome [67], or adults with a spinal cord injury [71].

Detailed psychometric properties were only reported for the quantitative measurement instruments. The most detailed results were available for questionnaires of the ICECAP-family. Both the *ICECAP-O* and the *ICECAP-A* were reported to have good construct [49, 51, 52, 64, 66], convergent [29, 53, 55, 58, 60, 62, 67, 70, 71] validity when compared to the EQ-5D instrument to measure generic health status, and discriminant validity [29, 55, 58, 67, 71]. The *ICECAP-O *and *ICECAP-A* further showed good test-retest reliability [29, 43, 57, 62, 77] and good internal consistency [29, 51, 58, 70]. In addition, the *ICECAP-A* was also found to be significantly responsive among adults with knee pain [66] and women with irritative lower urinary tract syndrome [67]. No psychometric properties were reported for the *ICECAP-SCM*, *ICECAP-FC* questionnaires. In the *OCAP* family, no details were available for the originally developed questionnaire [35]. The *OCAP-18* only yielded good construct validity when correlated with the EQ-5D-3L questionnaire [42]. The adaption of the *OCAP* for mental health showed good convergent validity [36, 74], internal consistency, and test-retest reliability [74], which was also confirmed for its German version [73]. With respect to the other questionnaires, Sacchetto et al. [38] reported good content and discriminant validity as well as internal consistency for the *ACQ-CMH*. The* CADA* questionnaire [40] reported good internal consistency for most questions, while the *Capability Measurement Tool for People with Chronic Pain* [39] did not report any psychometric properties.

### Overall capabilities, capabilities for health, and capabilities for PA

While some of the questionnaires focus on the overall capabilities to pursue one’s goals and being content with one’s own life (e. g. the *ICECAP* questionnaires [31–34]), others are concerned with more specific aspects, such as enjoying recreational time, political views, making friends, or areas relevant to this study, e. g. bodily health and integrity (e. g. *OCAP* questionnaires [35, 42, 74]). Some questionnaires focus on specific subsets of health enhancing factors, such as the *CADA* [40], which is concerned with capabilities for healthy diet and PA but does not measure overall capabilities for health or well-being. A similar pattern can be found for the qualitative tools: While Ndomoto et al. [24] focus on general capabilities for health, Abu-Zaineh et al. [44] explicitly deal with capabilities for health and self-management diabetes patients. Sauter et al. [25] is the only qualitative tool with a focus on capabilities for PA as a health-promoting factor.

Among the questionnaires, *CADA* [40] is the only one to directly measure capabilities for PA by specifically asking about resources (e.g. money to afford going to the gym) as well as environmental (e.g. indoor and outdoor PA spaces available), social (e.g. surrounding people are supportive of one’s PA) and individual (e.g. mental and physical health influencing PA) factors of influence. The other questionnaires do not specifically ask for capabilities to pursue PA or sports but at least partially address areas that can be considered relevant for health-enhancing PA, such as physical suffering (*ICECAP-SCM* [33]), bodily health or enjoyment of recreational activities (*OCAP* [35] and *OCAP-18* [42]). The qualitative tools do not explicitly address capabilities for PA. The only exception is Sauter et al. [25], which specifically asks for the individual (e. g. knowledge about PA), social (e. g. family and friends support) and environmental factors (e. g. offerings) that influence the opportunities of seniors in retirement homes to be physically active.

## Discussion

The aim of this review has been to give an overview of the current state of research on available tools to measure capabilities for health based on the approach originally developed by Sen and Nussbaum, with a special focus on identifying those potentially relevant for HEPA. The systematic search was able to identify capability measurement tools for health and HEPA using qualitative, quantitative, and mixed methods between 2008 and 2020. It has explored the main features and psychometric properties of the identified tools, as well as their past application to different age and target groups.

Despite the number of papers identified, it is interesting to note that the number of distinct tools reported remains limited. For instance, there is a total of eleven questionnaire-based tools, most of which are variations and adaptations of either the *ICECAP* or the* OPAC* questionnaire. It is noteworthy that, although there are variations of the above-mentioned questionnaires for the use among different target groups, there is no tool available to objectively and comprehensively measure all aspects of health-related capabilities, especially when considering that the approach was first published in 1985 [1], connected to well-being as early as 1993 [3], and has recently gained even more attention in the field of public health.

The analysis revealed a great degree of methodological variation regarding the development of the interview guidelines and questionnaires. Some studies approached the development from a more philosophical view and based their interview guideline [25] or questionnaire items [35] on Martha Nussbaum’s capability criteria [72]; others used an explorative approach, conducting focus-group [39, 40] or key-expert interviews [36, 42] to inductively develop their questionnaire. Another research group developed the questionnaire based solely on expert-group’s opinion [38]. While our results allow no conclusions about which method is more appropriate or valid, those choosing a tool for a specific health promotion project should consider whether its development method and target group fit the intended application context. The variety of the available tools suggest that measuring capabilities may generally be a rather context- and target group-specific undertaking and may always require adaptation to different contexts and target groups. However, as this impedes the comparability of studies that target capabilities for health, working towards the development of tools applicable to more than one context may seem necessary.

The analyzed questionnaires that were empirically tested showed a moderate to good validity, reliability and responsiveness among different groups and compared to other questionnaires, mostly variations of the EQ-5D well-being questionnaire (i. e. EQ-5D-3L). This approach, however, poses an important theoretical issue, as it seems to imply that capability measures are better if they have a higher degree of correlation to measures of well-being. But according to Sen, well-being is a combination of “achieved functionings” [3], which are linked to but by no means perfectly correlated to a person’s options (capabilities). To give an example, a person with a variety of options that may positively influence their health has the freedom to choose their eventual course of action and may actively decide *not* to realize a specific behavior. If we take the capability approach seriously, we must necessarily expect a considerable mismatch between functionings and capabilities and using this kind of validation approach appears as generally problematic. To validate such a measurement tool, a more comprehensive and thus perhaps more challenging approach might be necessary, e. g. by attempting to account for all individual, structural, and environmental opportunities as well as a target group’s resources to validate the instrument.

Another issue is that the number of items used to measure capabilities also varied considerably between questionnaires, i. e. between five items (*ICECAP-O/ICECAP-A*) and 104 items (*OCAP*). This raises the question whether all identified tools – even though they may have been validated – allow for measuring with the same accuracy. More research is required to investigate this, but in any case, health promoters interested in measuring capabilities will have to consider whether it will be feasible to administer the tool of their choice in practice, especially regarding those with a large number of items.

Most questionnaires were developed for a specific population group, e. g. adults (*ICECAP-A* [32], *OCAP/OCAP-18 *[35, 42], *CADA* [40]), older adults (*ICECAP-O* [31]) or people suffering from mental illnesses (*OxCAP-MH* [36]; *CQ-CMH* [37]). However, even the general population questionnaires were often validated using samples of vulnerable population groups (e. g. adults with dementia [58], diabetes and obesity [40], or post hospitalization [55]). This may have implications for both the applicability and validity of the results for the general populace.

Our findings seem to support the conclusions of a previous literature review by Helter et al. [7] that there remain important conceptual and methodological issues in the field of measuring capabilities. At the same time, our study adds a new perspective, as Helter et al. [7] investigated the use of tools for economic evaluation while our main focus has been on measuring change and health intervention effectiveness.

Our research was guided by the intention to identify suitable tools for measuring capabilities for PA across the life-course. However, only two of the identified measurement instruments explicitly address PA, i. e. the *CADA* questionnaire [40] and the interview-based tool by Sauter et al. [25]. However, *CADA* is not geared exclusively at PA but combines it with capabilities for healthy diet. In addition, it was developed for populations suffering from obesity rather than general populations. Similarly, Sauter et al.’s tool has a specific focus on senior citizens. In other questionnaires, only individual items might be considered relevant for PA, e. g. questions on bodily health [35, 36, 42]. Therefore, they cannot be applied to draw precise conclusions on PA capabilities of people.

However, this study is able to provide researchers and health promoters with a number of options for measuring capabilities that may be useful for the field of HEPA by adapting them accordingly.

All in all, our study shows that more research is needed to develop appropriate capability instruments for HEPA. First, these should focus on measuring PA and all its facets, including the individual (e. g. PA-related competence), social (e. g. social support for PA), and environmental (e. g. PA infrastructures and offers) conversion factors. Second, a future measure for capabilities should ideally be applicable to a broader range of different settings, populations, and age-groups, thus allowing for standardized and comparable assessments of PA intervention effectiveness.

As HEPA can be considered a functioning which is intended to be changed by interventions, a combination of measuring both capabilities and functionings (e. g. as done by Al-Janabi [34]) might be advisable in the field. This may help future researchers to identify effects of their interventions on both levels.

We were able to identify very context-specific measurement tools, which seems appropriate due to the context specific nature of the capability approach but is likely to impede the comparability of interventions effectiveness.

To strike a compromise between detailed but setting-exclusive tools and overly generic instruments, there might be a need for a framework for conceptualizing and measuring capabilities for health including our aim of health enhancing PA across the life-course, as it was done with the* ICECAP* measurement tool [79]. Such a framework is currently in preparation, with the intention to define a number of principles that will ensure a greater amount of comparison between age groups and settings while still allowing for the use of adapted instruments in different contexts (Till M, Gelius P, Abu-Omar K, Abel T: Using the capability approach in health promotion projects: a framework for implementation, Under review).

Despite our best of efforts, this study has some limitations which need to be borne in mind when interpreting its results and drawing conclusions. First, due to the heterogeneity of the tools identified, comparing individual instruments with each other was difficult, and it was therefore not possible to recommend a single tool that, in general, could be considered to be particularly appropriate. For the same reason, a more systematic quality assessment of the primary studies, as required by the PRISMA checklist, was not possible. Further, as we only included studies on psychometric properties that came up in our initial systematic search but did not perform a second search for psychometric property measurements for all identified quantitative tools, the results shown in this paper may miss some studies. All in all, however, we are confident that this review provides a good initial overview in an innovative and increasingly relevant area of research. Having been conducted on a large number of databases and employing an additional hand search, it presents details on different types of instruments that may guide the selection of appropriate tools for specific purposes in future research projects.

## Conclusion

This systematic review has shown that there is a large variety of measurement tools available which address different aspects of capabilities, target groups or contexts. Until now, there is no golden standard on how to measure capabilities for health and therefore also none for PA. The available tools vary substantially regarding their underlying assumptions, focus on capabilities, properties (e. g. language, number of items), development processes, measurement approaches, and addressees. Most of the quantitative tools have been empirically shown to be valid, reliable and responsive, but the methods employed for validation invite skepticism as to whether all instruments truly measure capabilities and/or do so in a meaningful way. At this point in time, it is not possible to recommend a single tool for general use, and health promoters may want to choose carefully or even consider adapting a tool to their specific needs. Our findings may help inform researchers about available measurement tools that represent different options on how to measure capabilities for health and well-being, and which can be used as references for the future development of a measurement tool for capabilities for health enhancing PA.

Our findings thus seem to echo Sen’s own concerns about the empirical difficulties of operationalizing the capability approach [1, 80], as well as those of other researches who have demurred that the multidimensional, context-dependent, and normative nature of the approach can pose problems for operationalization [81–83].

These difficulties notwithstanding, the Capital4Health consortium, under whose auspices this review was conducted, is planning to contribute to the further development of capability measurement in health promotion and PA intervention research.

## Data Availability

All data used to derive the study findings are included in this published article.

## Abbreviations

ADL: Activities of daily living
ADRQL: Alzheimer’s disease related Quality of life
BPRS: Brief Psychiatric Rating Scale
BPRS: Brief Psychiatric Rating Scale
C: Comparison
CQ-CMH: Capability Questionnaire for Community Mental Health
CTM-3: 3-Item Care Transition Measure
D: Development
DASS-D: Depression Anxiety Stress Scales
EFA: Exploratory factor analysis
FG: Focus group
GAF: Global Assessment of Functioning
HEPA: Health enhancing Physical activity
ICC: Intra-class correlation coefficient
ICECAP-A: ICEpop CAPability measure for adults
ICECAP-FC: ICEpop CAPability and Functioning measure
ICECAP-O: ICEpop CAPability measure for older people
ICECAP-SCM: ICEpop CAPability measure for supportive care
K6: Kessler Psychological Distress Scale
M: Mean
M: Measurement
OCAP: Oxford Capability Questionnaire
OLS: Ordinary least square
OXCAP-MH: Oxford Capability Questionnaire for Mental Health
PA: Physical Activity
PHQ-8: Patient Health Questionnaire depression scale
RAS: Recovery Assessment Scale
RT: Researcher Team
SEM: Structural equation modeling
SIX: Objective Social Outcomes Index
V: Validation
WB: Well-Being
